# Supplementary material for: Evidence for domain-general arousal from semantic and neuroimaging meta-analyses reconciles opposing views on arousal
Source: Proc Natl Acad Sci U S A. 2025 Feb 3;122(6):e2413808122. doi: 10.1073/pnas.2413808122 (PMC11831115; doi:10.1073/pnas.2413808122)
Supplement: Supplementary file 1 — Appendix 01 (PDF) [file pnas.2413808122.sapp.pdf]

Supporting Information for
Evidence for domain-general arousal from semantic and neuroimaging meta-analyses reconcile opposing views on arousal.

Magdalena Sabat[1,2]*, Charles de Dampierre[3], Catherine Tallon-Baudry[1]**

Corresponding authors: * Magdalena Sabat, ** Catherine Tallon-Baudry
Email: * magdalena.sabat@ens.psl.eu, ** catherine.tallon-baudry@ens.psl.eu,

This PDF file includes:

Supplementary Figures S1 to S5
Tables S1 to S5

Detailed list:

Supplementary Figure 1 – Post-hoc validation of cluster number selection using the silhouette method.
Supplementary Figure 2 – Semantic network for different granularities
Supplementary Figure 3 – Meta-analyses as in Main Fig 2, unthresholded
Supplementary Figure 4 – Meta-analyses as in Main Fig 2, region of interest included
Supplementary Figure 5 – Neuroimaging meta-analyses for 1500, 2000, 2288 terms in semantic analysis

Supplementary Table 1 – Guidelines for term inclusion
Supplementary Table 2 – Most frequent terms and terms related to tasks and paradigms
Supplementary Table 3 – Terms with largest inside degree centrality
Supplementary Table 4 – Dictionary of terms used for physiological measures
Supplementary Table 5 – Percentage of abstracts with the terms “phasic” or “tonic”

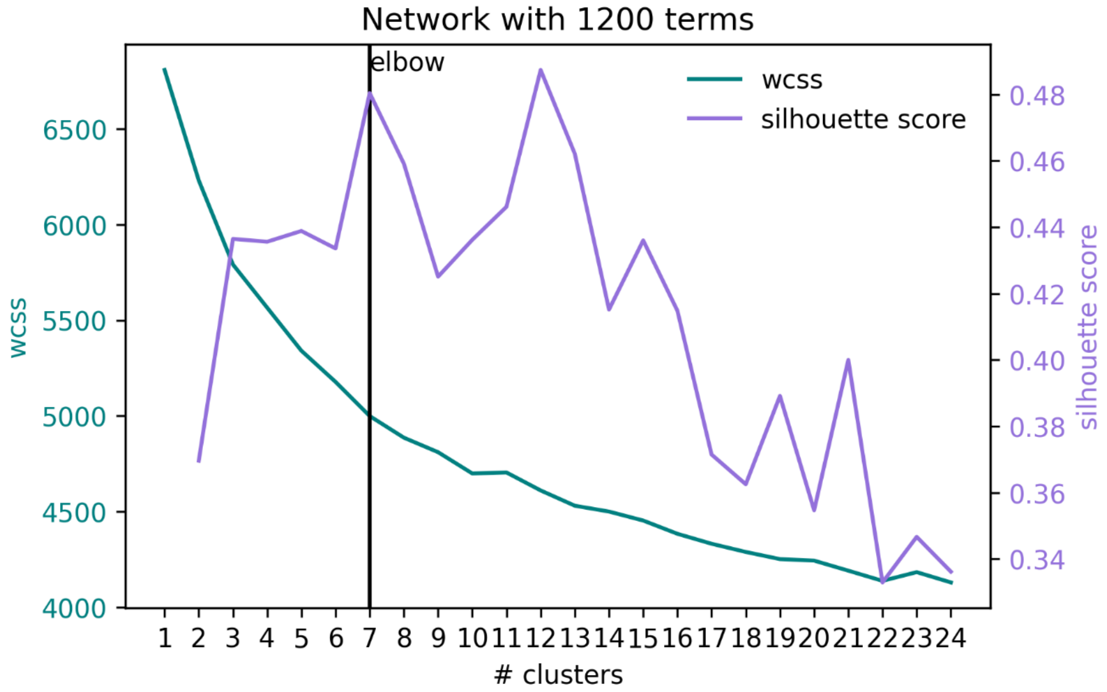

Supplementary Figure 1. Post-hoc validation of cluster number selection using the silhouette method. To validate the number of clusters in the semantic analysis we performed a post-hoc validation using the silhouette method. Both the elbow method performed on the within-cluster sum of squares (wcss, the left y-axis) and the silhouette analysis of the silhouette score (the right y-axis) return 7 clusters as optimal number of clusters. The silhouette methods shows a second peak at 12 clusters.

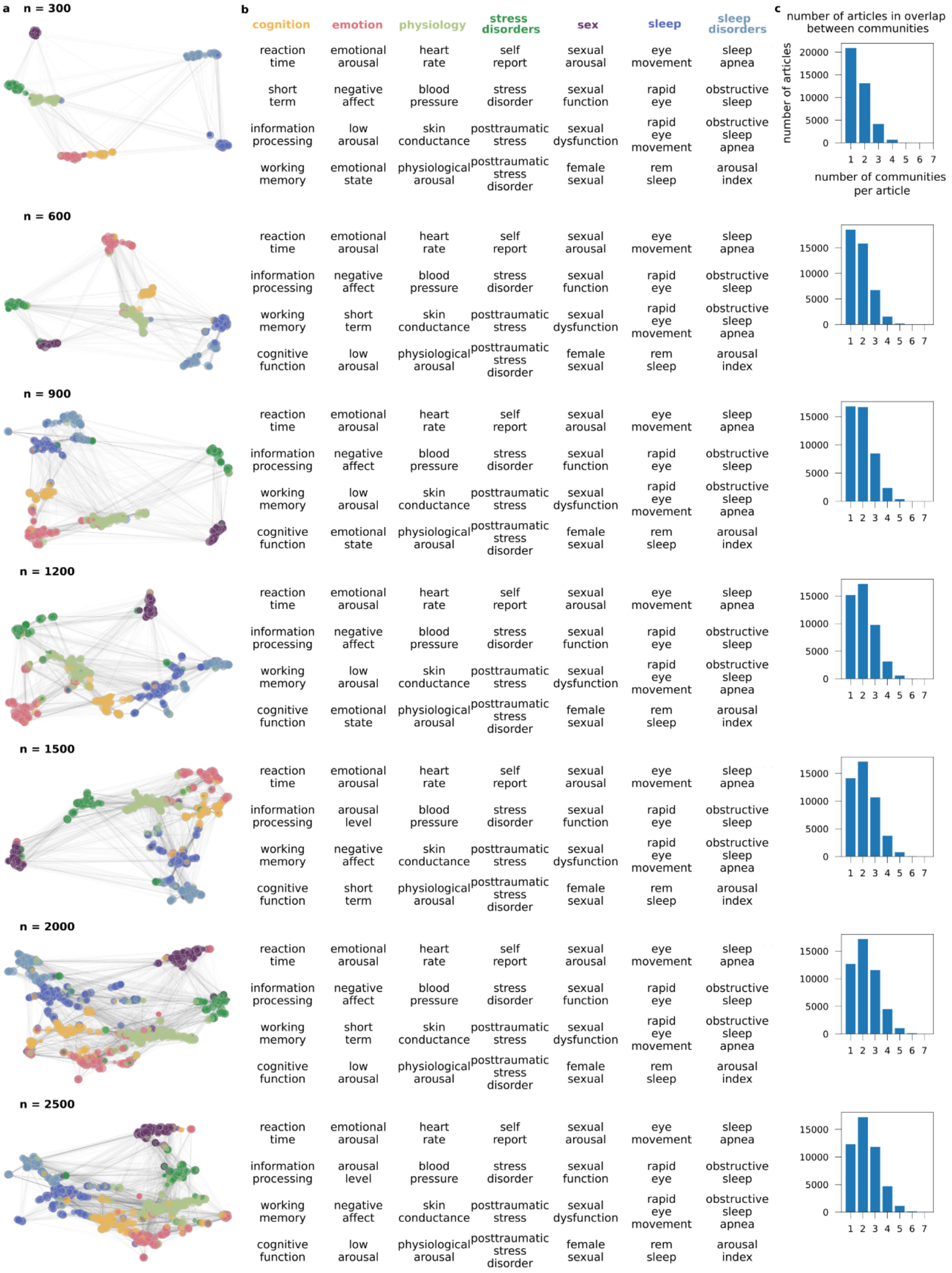

Supplementary Figure 2. The organization of the semantic network remains stable when varying the number of terms included in the semantic analysis. We conducted the semantic analysis on a number of terms varying from 300 to 2285. The number of clusters and their semantic contents remains stable. **A.** Semantic network and clusters. **B.** The four most occurring

terms in each cluster. **C.** Histograms describing the number of semantic clusters each article is associated with.

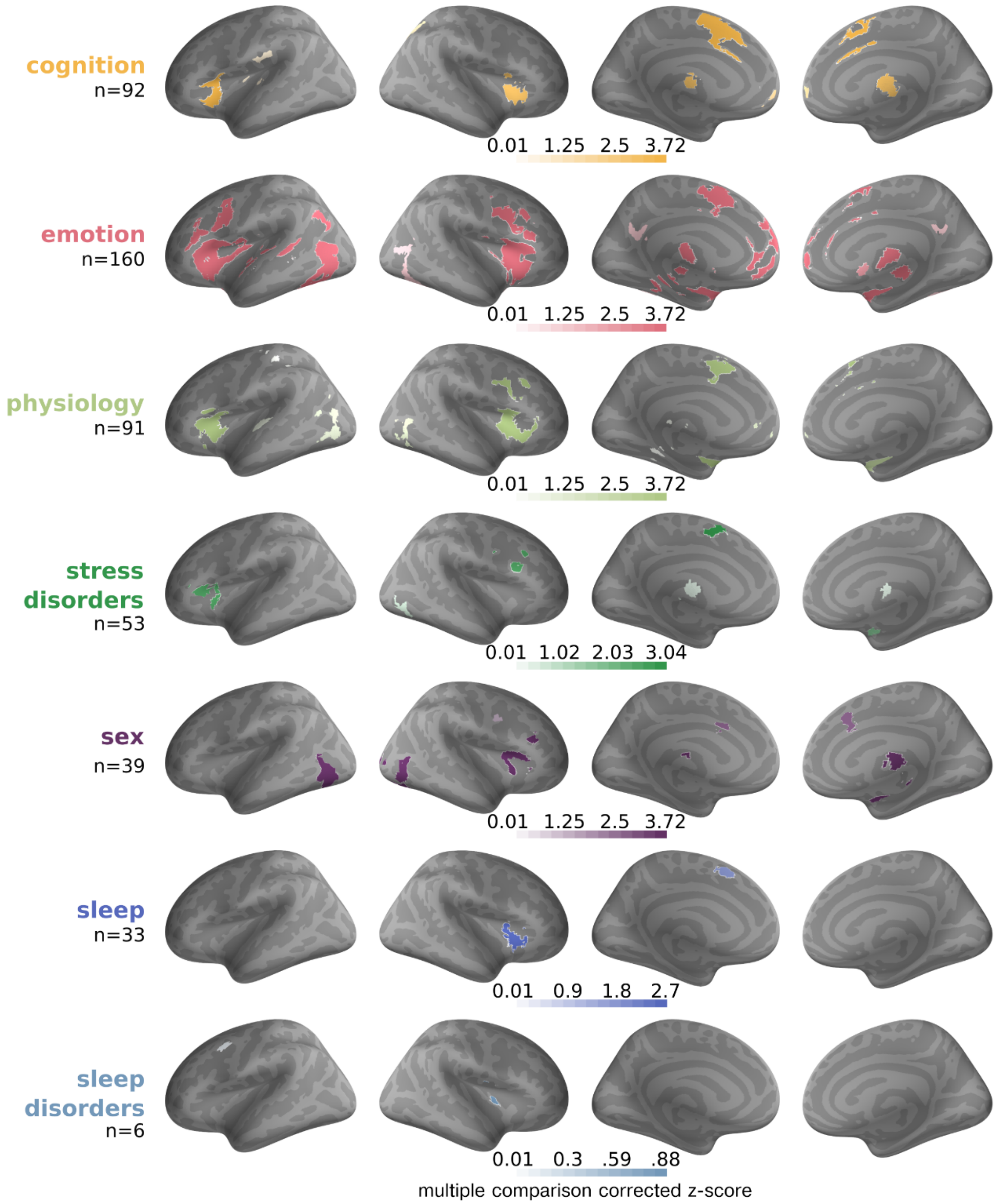

Supplementary Figure 3. Unthresholded results of meta-analyses (z-score, corrected for multiple comparisons) of the neuroimaging meta-analyses reported in Figure 2. Unthresholded data do not reveal more overlap in the cortical arousal network.

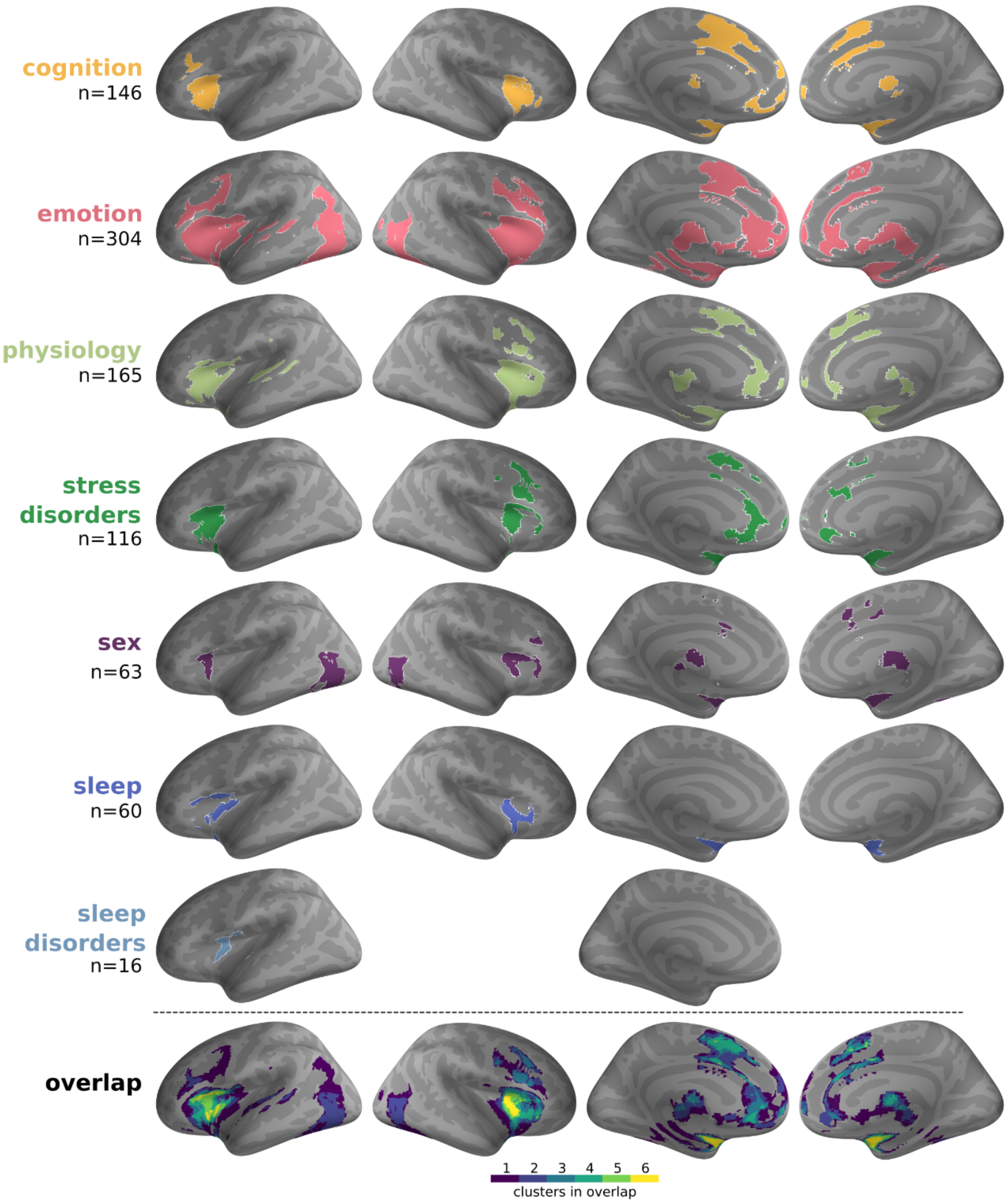

Supplementary Figure 4. Results of the neuroimaging meta-analysis in the network of 1200 terms with articles that used a priori region of interest analysis. Including fMRI studies that used a priori region of interest analysis in the meta-analysis maintains the overlap in the left and right anterior insula and preSMA, but reveal an additional cluster in the amygdala bilaterally.

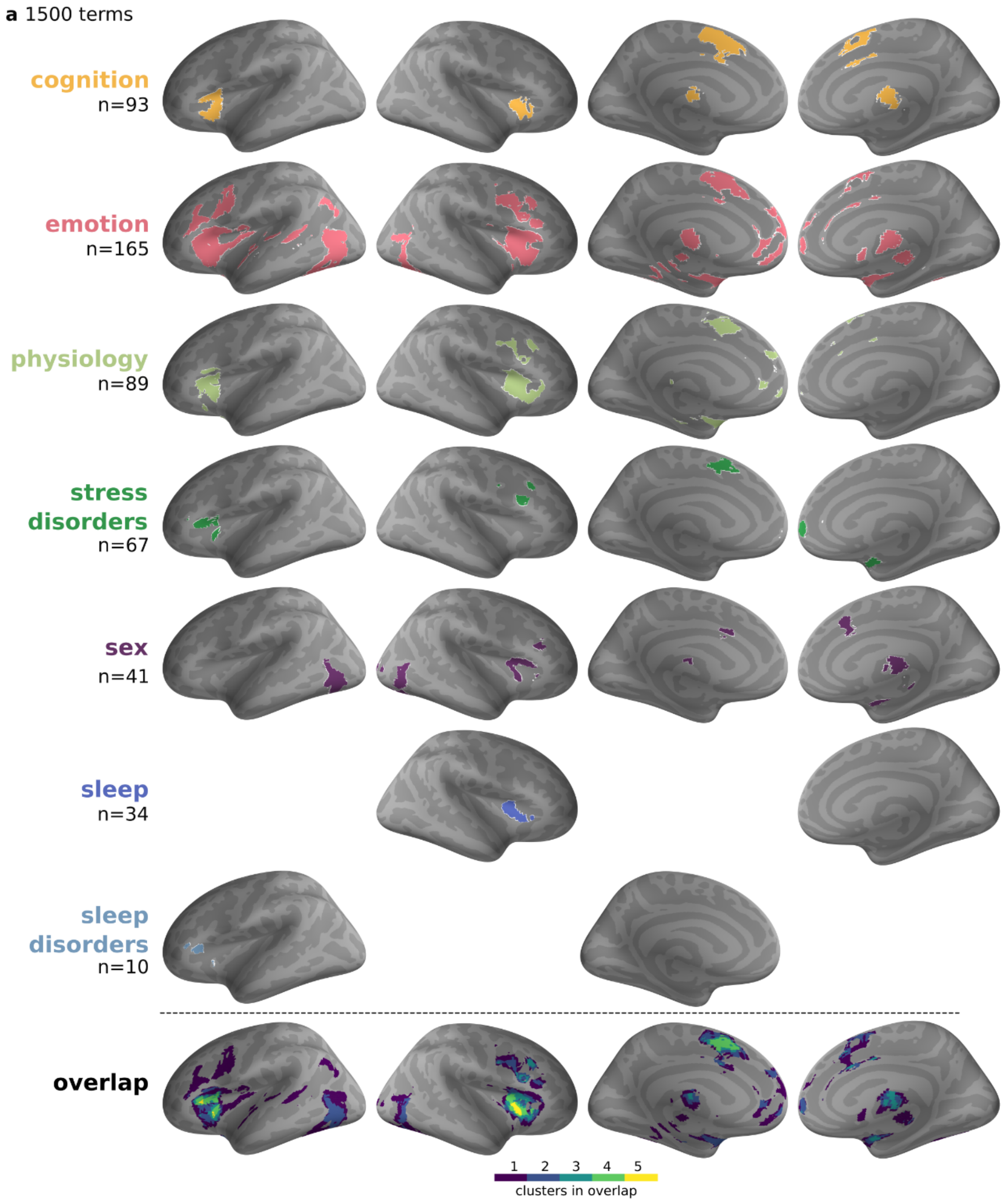

a 1500 terms
cognition
n=93
emotion
n=165
physiology
n=89
stress
disorders
n=67
sex
n=41
sleep
n=34
sleep
disorders
n=10
overlap
1 2 3 4 5
clusters in overlap

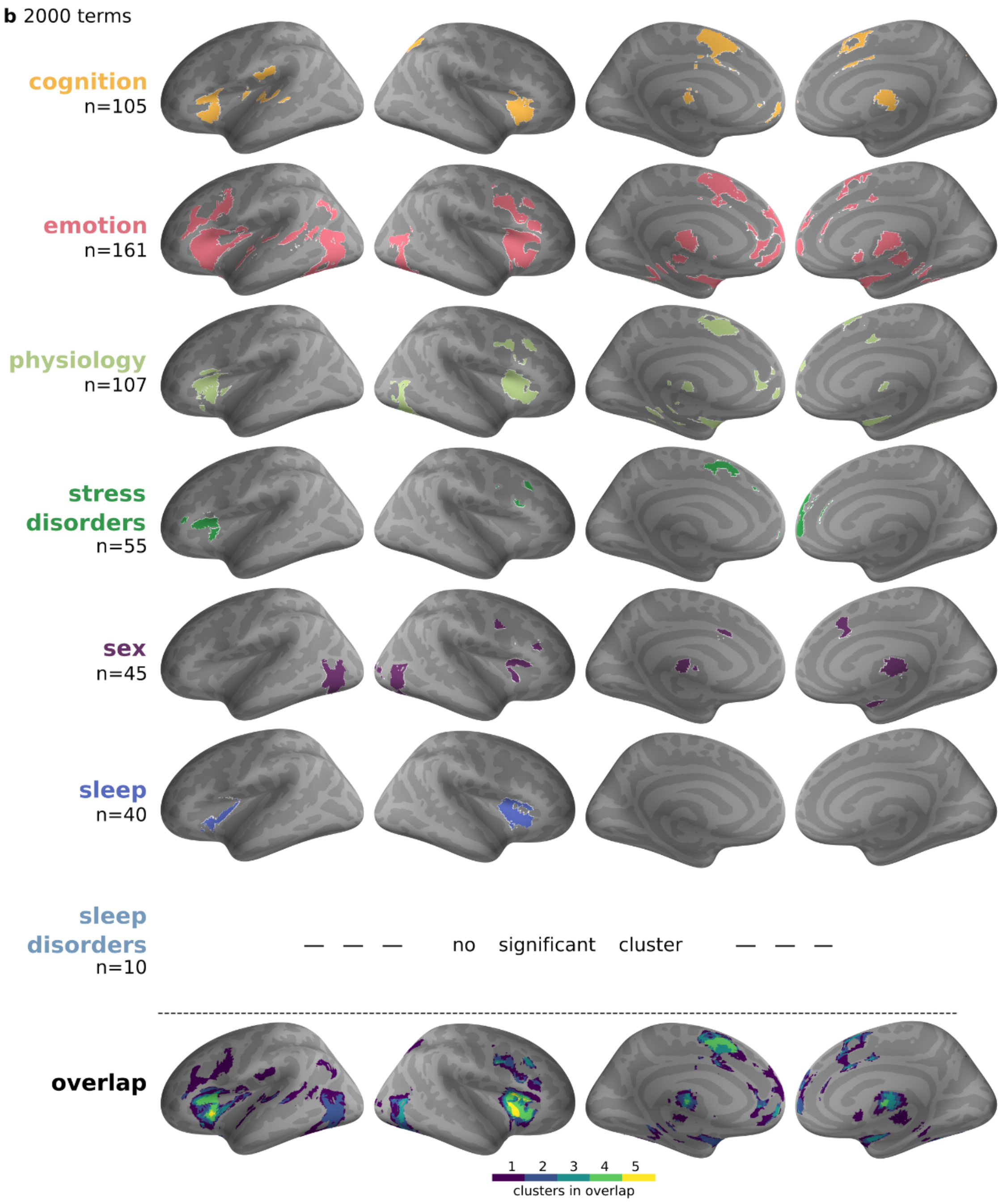
b 2000 terms
cognition
n=105
emotion
n=161
physiology
n=107
stress disorders
n=55
sex
n=45
sleep
n=40
sleep disorders
n=10
— — — no significant cluster — — —
overlap
1 2 3 4 5
clusters in overlap

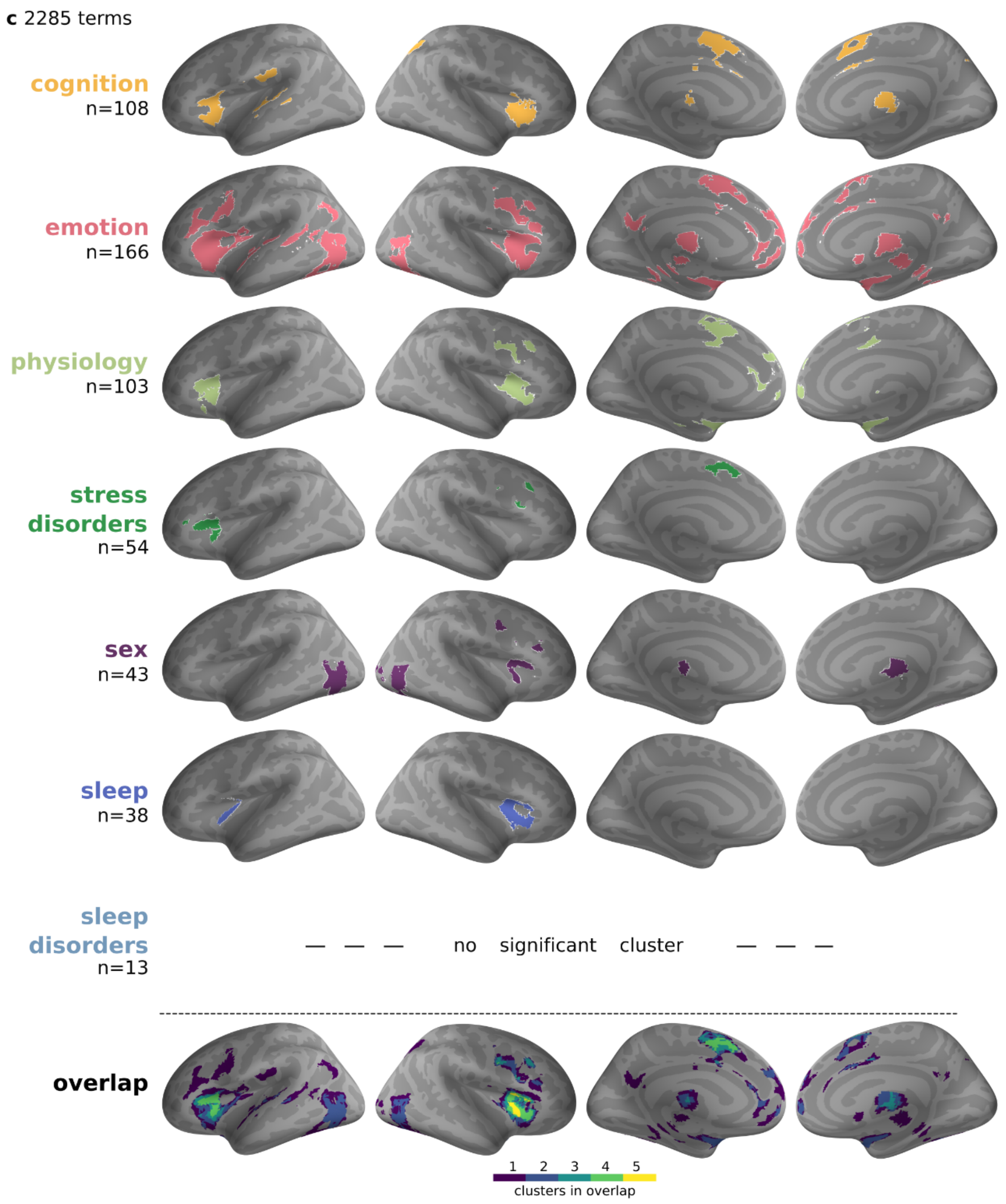

Supplementary Figure 5. Brain-imaging results are stable for different numbers of terms in the semantic analysis. Results of the neuroimaging meta-analysis across the number of terms (1500, 2000, 2285) included in the semantic analysis: **A.** 1500 terms, **B.** 2000 terms, **C.** 2285 terms. Across the number of terms used in the semantic analysis only small differences in the overlap are found. The cortical arousal network, composed of left

and right anterior insula and preSMA is consistently found to be the overlap foci across all analyses.

Supplementary Table 1. Inclusion/Exclusion criteria - guidelines for term selection. We excluded terms related to, or terms that contained a word related to, the categories listed below.

**Guidelines for exclusion (in quotes see literal examples)**		
	literal example	exception/ comment
Non-specific general research related words:		
Items describing statistical method, procedures and results like methods, descriptives, significance	“regression”, “SD”, “SVD”, “convolutional neural network”, “significant improvement”	due to ambiguity keep “neural network”
Items describing general study results	“behavioral results”,“Randomized trials”	
Items describing species & species subgroup	“rat model”, “human”, “C.elegans”,“heterosexual men”, “older adults”, “OSA patients”	“depressive patients” should be excluded but “depression” should be kept
Items describing subject pool and terms describing non-specific treatment of the experimental group	“control group”, “test group”, “treatment group” “treatment options”, “clinical variables”, “clinical features”	“Overweight group” should be excluded but “obesity” or “body mass” should be kept
Items describing change	“faster responses”, “treatment effect”,” increased result” “higher arousal”	KEEP terms describing the potential variable (“reaction time”, “FSFI scores”,” age effects”) or measurement tools (“fMRI”, “EEG” etc.)!

Items describing general experimental design	“twin study”, “crossover study”, “task demand”	KEEP terms describing experimental material, like task (ex. “stroop task”) or potential stimuli used (ex. “neutral scenes”, “video clips” etc.)!
Items describing Editors, Journals, International/Scientific/Academic societies	“Sexual Medicine”, “Neurophysiology”, “International Society”	
**Others:**		
Items that have little semantic significance or terms that don’t refer to any specific variables, for example	units (“micro gram”,” celsius degrees”),” last decade”, “large number”, “peak amplitude”, “frequency and duration”, “treatment approach”, “first night”, “greater risk”, “objective measures”, “Similar results”, “previous research”, “relative contribution”, “index score, “function scores”	importantly exclude “index score”, “function scores” as they’re not referring to any specific variable but keep “FSFI score” or “arousal index” as they refer to specific variables
Countries or other Geographical locations	Hong Kong, USA etc,	

Supplementary Table 2. Table with the five most frequent terms in each semantic community and their respective number of occurrences, and all terms related to tasks and paradigms in each community.

Semantic Community	Five most frequent terms, occurrence	All task and paradigm related terms, occurrence
cognition	'reaction time': 1487,<br>'information processing': 762,<br>'working memory': 630,<br>'cognitive function': 599,<br>'attention deficit': 520	'task performance': 463,<br>'memory task': 357,<br>'vigilance task': 350,<br>'cognitive task': 325,<br>'time task': 247,<br>'reaction time task': 234,<br>'discrimination task': 201,<br>'attention task': 180,<br>'stroop task': 172,<br>'detection task': 132,<br>'task difficulty': 126,<br>'performance task': 120,<br>'oddball paradigm': 119,<br>'psychomotor vigilance task': 114,<br>'motor task': 113<br>'task condition': 112,<br>'oddball task': 99,<br>'search task': 78,<br>'tracking task': 74,
emotion	'emotional arousal': 1445,<br>'negative affect': 805,<br>'low arousal': 730,<br>'emotional state': 706,<br>'emotional response': 703	'recognition task': 173,<br>'learning task': 153,<br>'decision task': 107,<br>'recall task': 79<br>'lexical decision task': 76,
physiology	'heart rate': 4275,<br>'blood pressure': 2052,<br>'skin conductance': 1616,<br>'physiological arousal': 1211,<br>'arousal level': 975	'conditioning paradigm': 125<br>'arithmetic task': 121,<br>'stress task': 95,<br>'mental arithmetic task': 94,
stress disorders	'self report': 1627,<br>'stress disorder': 1487,<br>'posttraumatic stress': 1103,<br>'posttraumatic stress disorder': 1002,<br>'anxiety disorder': 866	'avoidance task': 119

sex	'sexual arousal': 2084,<br>'sexual function': 1363,<br>'sexual dysfunction': 1340,<br>'female sexual': 1334,<br>'sexual desire': 861	None
sleep	'eye movement': 2092,<br>'rapid eye': 1637,<br>'rapid eye movement': 1635,<br>'rem sleep': 1439,<br>'slow wave': 1198	None
sleep disorders	'sleep apnea': 1581,<br>'obstructive sleep': 1524,<br>'obstructive sleep apnea': 1283,<br>'arousal index': 1029,<br>'risk factors': 947	None

Supplementary Table 3. For each semantic community, the five terms with the largest number of connections within the community and their inside degree centrality.

Semantic Community	Term, Degree inside
Cognition	Reaction time, 89<br>Choice reaction, 37<br>Cognitive performance, 36<br>Choice reaction time, 34<br>Task performance, 32
Emotion	Emotional stimuli, 88<br>Neutral pictures, 54<br>Emotional pictures, 52<br>Unpleasant pictures, 44<br>Negative picture, 43
Physiology	Heart rate, 203<br>Blood pressure, 131<br>Rate variability, 85<br>Hear rate variability, 85<br>Cardio-vascular response, 80
Stress disorders	Stress disorder, 81<br>PTSD symptom, 68<br>Post-traumatic stress, 66<br>Post-traumatic stress disorder, 65<br>Trauma exposure, 50
Sex	Sexual dysfunction, 95<br>Female sexual, 95<br>Sexual desire, 84<br>Sexual function, 84<br>Female sexual dysfunction, 78
Sleep	Rapid eye, 117<br>Rapid eye movement, 116<br>Eye movement, 116<br>REM sleep, 96<br>nREM sleep, 96
Sleep disorders	Obstructive sleep, 94<br>Sleep apnea, 91<br>Obstructive Sleep Apnea, 86<br>Apnea hypopnea, 73<br>Hypopnea index, 70

Supplementary Table 4. Dictionary of terms used to find physiological measures in the texts.

**measure**	**Terms searched for**
heart	['heart-rate', 'heart rate', 'hrv', 'interbeat interval', 'IBI', 'inter-beat interval', 'cardiac rate', 'cardiac response']
skin conductance	['skin conductance', ’skin resistance’, 'electrodermal', 'galvanic skin response', 'SCR', 'GSR', 'EDA']
pupil	['pupil', 'pupillography', 'pupillometry', 'oculography', ‘oculometry’, ’eye tracking’, ‘eye-tracking’]
respiration	['respiration', 'respiratory', 'breathing', 'inhalation', 'exhalation', 'ventilation', 'ventilatory']
blood pressure	['blood pressure', ’systolic pressure'’, 'diastolic pressure']

Supplementary Table 5. Percentage of abstracts containing term 'phasic' or 'tonic' in each semantic cluster in the network composed of 1200 terms.

	tonic	phasic	difference
sex	1.03	0.97	0.06
sleep disorders	1.87	1.83	0.04
emotion	1.5	1.56	-0.06
cognition	2.59	2.88	-0.28
sleep	3.17	2.98	0.19
physiology	2.33	2.23	0.1
stress disorders	1.19	1.09	0.09
